# Plasma-Enhanced Spatial Atomic Layer Deposition on 2D and 3D Surface Topologies: The Case of Amorphous and Crystalline TiO_2_

**DOI:** 10.1021/acs.jpcc.4c08281

**Published:** 2025-01-31

**Authors:** Mike van de Poll, Jie Shen, James Hilfiker, Marcel Verheijen, Paul Poodt, Fieke van den Bruele, Wilhelmus Kessels, Bart Macco

**Affiliations:** † Department of Applied Physics and Science Education, Eindhoven University of Technology, 5600 MB Eindhoven, The Netherlands; ‡ TNO/Holst Centre, High Tech Campus 31, 5656 AE Eindhoven, The Netherlands; § J.A. Woollam Co., Inc., 311 South Seventh Street, Lincoln, Nebraska 68508, United States; ∥ SparkNano B.V., Esp 266, 5633 AC Eindhoven, The Netherlands

## Abstract

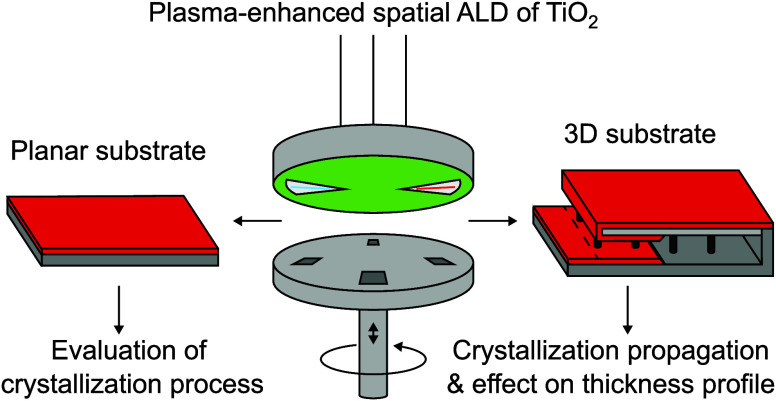

Plasma-enhanced spatial ALD (PE-s-ALD) is an interesting technique for high-volume manufacturing of thin films at low-temperature. This technique is particularly appealing for conformal depositions on 3D surfaces for various applications, such as optical coatings, electrolyzers, and batteries. However, various crystallization and growth effects can influence the final film profile and properties, and understanding these effects and their interplay is key. This study investigates the complex growth mechanism of TiO_2_ using PE-s-ALD. TiO_2_ films are deposited on both planar and 3D substrates, while systematically varying the number of cycles, deposition temperature, and exposure times. Thickness, crystallinity, and composition are determined as a function of depth inside the structures. Conditions that result in the anatase phase on a planar surface only partially form this phase inside 3D structures, with the deepest part of the film being amorphous. This partial crystallization is ascribed to the film thickness inside the 3D structure gradually dropping below the critical thickness for crystallization. In turn, the partial crystallization is shown to have a significant effect on the resulting thickness profile, due to a difference in growth per cycle between the two phases. A framework of the interplay between effects is proposed, offering insights that enable better control of crystallinity and thickness throughout the entirety of coated surfaces of 3D structures by PE-s-ALD. Additionally, the recombination probability of oxygen radicals during this atmospheric-pressure PE-s-ALD process at 200 °C is determined to be 3 × 10^–5^. This value is similar to low-pressure PE-ALD, indicating that differences in conformality between the two types of ALD are not the result of differences in recombination probability, but rather of differences in initial radical density and diffusion behavior.

## Introduction

Atomic layer deposition (ALD) is a vapor-phase deposition technique that utilizes self-limiting surface reactions to deposit high-quality thin films with excellent thickness control, uniformity, and conformality.^1−3^ However, temporal separation of precursors by sequential dosing of precursor and purging of the vacuum chamber makes conventional ALD relatively slow (typically a few nm per hour). Therefore, spatially separating the precursors by using inert gas curtains, while moving the substrate through the different reaction zones, is preferred for ample applications.^4^ This so-called spatial ALD (s-ALD) enables high throughput, low costs, and the use of large area substrates, while it allows for atmospheric-pressure processing conditions, eliminating the need for expensive vacuum equipment.^5^ Additionally, the use of plasma as co-reactant is appealing, since it facilitates enhanced growth rates and processing at low temperatures.^6−8^ Furthermore, recently we have shown that plasma-enhanced s-ALD can prepare highly conformal metal oxide films in 3D structures, owing to the high radical density in the atmospheric-pressure plasma source.^9^

In this work, the deposition of TiO_2_ thin films into high-aspect ratio structures by plasma-enhanced spatial ALD is investigated. TiO_2_ thin films have many applications, such as photocatalysts,^10−12^ gas sensors,^13^ high-*k* oxides,^14,15^ and optical coatings.^16^ Plasma-enhanced spatial ALD is an appealing deposition method for these applications, since a significant part of them require coating over large areas, often on 3D topologies to further increase the surface area, as well as low deposition temperatures. ALD processes of TiO_2_ are relatively established and thoroughly investigated, with a wide range of precursors and co-reactant combinations having been reported.^17^ Moreover, TiO_2_ is also an interesting material from a processing perspective for various reasons. TiO_2_ can be grown as an amorphous film or in various crystal phases, i.e., anatase, rutile, or brookite. Its physical and chemical properties are highly phase dependent, hence understanding of the growth mechanism to tailor the film crystallinity to the specific application is essential. ALD studies have shown that its phase is influenced by the processing conditions and the film thickness.^18^ In general, a certain deposition temperature is required to grow a crystalline film, but the film is typically initially amorphous, even above the crystallization phase transition temperature. Once a temperature-dependent critical film thickness of an amorphous TiO_2_ film is reached, not only will the subsequently deposited material be crystalline, but also the previously deposited amorphous material will crystallize.^19,20^ The growth per cycle (GPC) during ALD is moreover highly phase dependent, with a significantly enhanced GPC on crystalline surfaces with respect to amorphous surfaces.^21−23^ Furthermore, TiO_2_ is a UV-sensitive material and exposure to photons above the bandgap of the material has been observed to enhance the GPC.^24^ All of these intricacies act simultaneously during (plasma) ALD of TiO_2_ on planar substrates. Additional questions regarding conformality arise when depositing inside 3D substrates. Such questions include the interplay between crystallinity and thickness throughout the 3D structures, and the influence of UV radiation, originating from the plasma, on the growth on exposed and shielded parts of the structure. While conformality is often thought of purely in terms of film thickness throughout 3D structures, it is also important to consider the uniformity of film properties throughout the structures.

In this work, the interplay between these effects is studied during PE-s-ALD of TiO_2_. Depositions were performed systematically on planar and 3D substrates, while independently varying the number of ALD cycles, deposition temperature, and rotational frequency of the substrate table. Varying the latter results in different precursor and plasma exposure times, which are intrinsically coupled in s-ALD. The film thickness, crystallinity, surface morphology, and composition were determined on planar films and as a function of depth in the 3D structures. From this, the crystallization process, conformality in terms of thickness and crystallinity, and the oxygen radical recombination probability at 200 °C were examined, similarly as done previously for PE-s-ALD at 100 °C and for temporal PE-ALD at various temperatures.^9,25^ Overall, this work shows that though highly conformal deposition of TiO_2_ is possible, detailed understanding of the interplay of growth mechanisms that govern TiO_2_ deposition is essential to control conformality in terms of both thickness and film properties.

## Experimental Section

**Figure 1:**
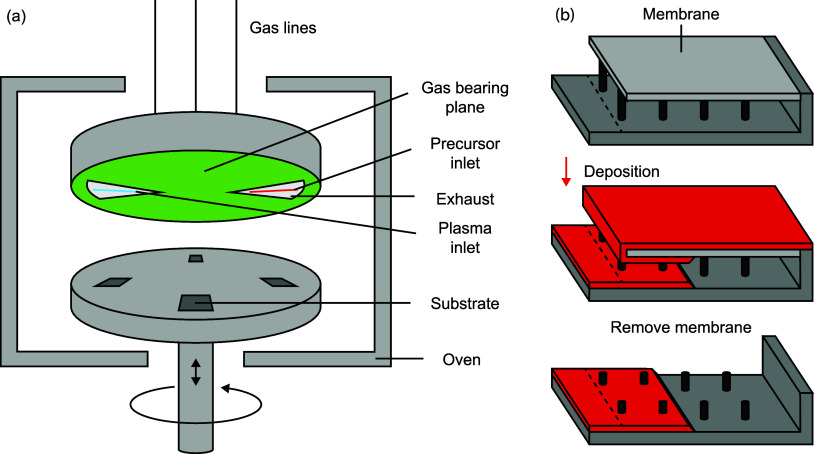
Schematic overview of experimental setup. (a) Rotary s-ALD reactor used to deposit TiO_2_ films on planar substrates and PillarHall chips. (b) Cross-section of a lateral high-aspect-ratio structure present on the PillarHall chip. The dashed line indicates the start of the trench.

TiO_2_ depositions were performed on a laboratory s-ALD reactor with a rotating substrate table (Figure 1a), described in more detail by Poodt et al.^26^ The deposition head was equipped with a remote dielectric barrier discharge (DBD) plasma source from SparkNano B.V., similar to the one described by Creyghton et al.^27^ By rotating the substrate table, samples pass sequentially through the precursor, purge, plasma, and second purge zones to complete an ALD cycle. Tetrakis(dimethylamino)titanium (TDMAT, Ti(NMe_2_)_4_, bubbler temperature = 40 °C) was used as the Ti-precursor and an N_2_–O_2_ plasma (2% O_2_, 50 V applied voltage) was used as co-reactant. Our standard condition used a deposition temperature of 200 °C, 420 ALD cycles, and a 2 rpm rotation frequency. From this standard condition, the temperature, number of cycles, and substrate rotation frequency were varied independently. During each deposition, both a planar Si substrate and a PillarHall chip were present in the reactor. PillarHall chips (Chipmetrics Ltd.) are Si test chips containing lateral high-aspect-ratio (LHAR) trench structures (Figure 1b) with a height of 500 nm and various lengths up to 5000 μm.^28,29^ These chips are specifically designed for conformality studies, where one of the trench walls can be removed with sticky tape after deposition to analyze the deposited film. The length of the LHAR structure characterized in this work is 1000 μm, corresponding to an aspect ratio of 2000.

Measuring thickness profiles within the LHAR structure was an essential part of these experiments, while doing so with high spatial resolution is not trivial. However, such thickness profiles were obtained by performing line measurements using a J.A. Woollam Small-Spot size (25 × 40 μm^2^) RC2 spectroscopic ellipsometer (SE). Each measurement was performed over a spectral range of 0.8–5.9 eV. The refractive index was found to vary throughout the trench. To obtain reliable thickness values while accounting for changes in optical constants, an optical model with a shifting-composition material file was used, based on Kramers–Kronig consistent B-spline functions fitted to several regions in the structure. This method was also used in our earlier publication, and more details can be found there and in its Supporting Information.^9^

The surface morphology was studied with atomic force microscopy (AFM) using a Bruker Dimension Icon, and scanning electron microscopy (SEM) using both a ZEISS Sigma and JEOL JSM-7500FA with accelerating voltage for imaging of either 1 or 2 kV. Crystallinity of the films was analyzed using X-ray diffraction (XRD) and Raman spectroscopy, using a Bruker D8 DISCOVER system with Cu Kα (λ = 1.54060 Å) radiation and a Renishaw inVia confocal Raman microscope, respectively. Because of the small probing radius (∼10 μm) of the latter, line-scan Raman measurements could be used to accurately determine the crystallinity as a function of depth in the LHAR structures. Similarly, the spatially resolved chemical composition throughout the films was determined by performing X-ray photoelectron spectroscopy (XPS) line-scan measurements using a Thermo Scientific K-Alpha XPS system with monochromated Al Kα source (λ = 8.3386 Å) and spot size of 30 × 30 μm^2^. Finally, cross-section transmission electron microscopy (TEM) images were taken to visualize the crystal structure of the films using a JEOL ARM 200F Transmission Electron Microscope, probe corrected, equipped with a 100 mm^2^ Centurio SDD EDX detector, operated at 200 kV.

## Results

### Deposition on Planar Substrates

**Figure 2:**
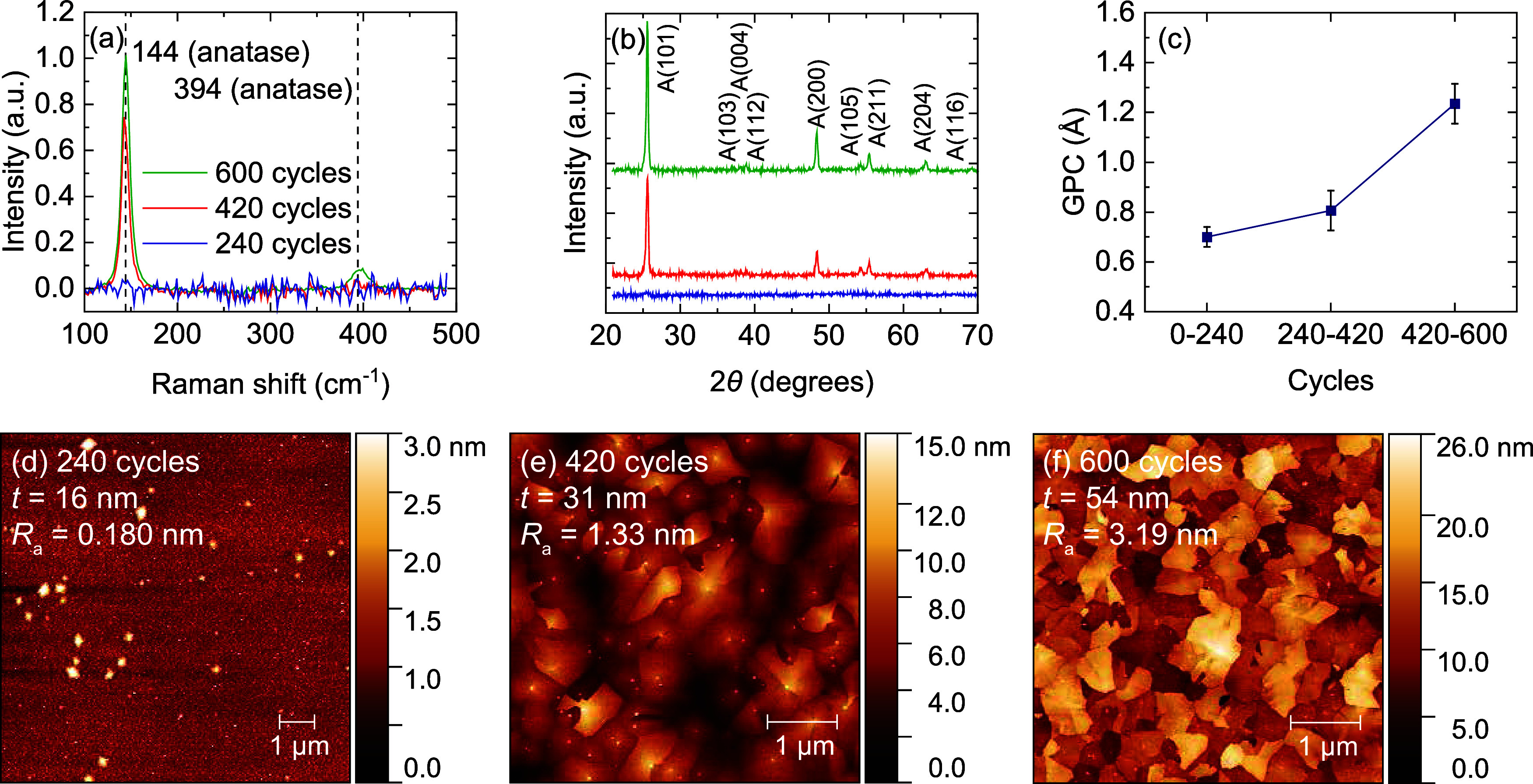
(a) Raman spectra normalized for film thickness, with dashed lines indicating characteristic peak positions for anatase TiO_2_, (b) grazing-incidence XRD diffractograms with offset lines for clarity, (c) average GPC determined by spectroscopic ellipsometry measurements on three separate samples, and (d–f) AFM images of PE-s-ALD TiO_2_ on planar Si substrates at deposition temperature of 200 °C and substrate rotation frequency of 2 rpm, for 240, 420, and 600 ALD cycles. Film thicknesses, shown in (d–f), were determined by spectroscopic ellipsometry.

The crystal phase of the films deposited on planar Si wafers, with 240, 420, and 600 ALD cycles (deposition temperature = 200 °C, rotation frequency = 2 rpm), has been determined using Raman spectroscopy (Figure 2a) and grazing-incidence XRD (Figure 2b). Both techniques show the transformation from amorphous structure at 240 cycles to the anatase structure at 420 cycles. The crystallization process continues up to 600 cycles, as can be seen from the Raman intensity, which increases even though it is normalized for the film thickness. No other crystal phases are present, as expected, since the rutile phase is typically only formed at deposition temperatures of 300 °C or higher.^30,31^ The preferred crystalline orientation was determined to be <101> from an additional gonio XRD measurement (Figure S1 in the Supporting Information).

The final thicknesses of the TiO_2_ films were determined using SE and compared with each other to obtain the growth per cycle (GPC) in different stages of the process (Figure 2c). While a broad range of GPC values are reported in literature for the TDMAT/O_2_ plasma process (i.e., 0.35–2.5 Å at 200 °C), the values presented here are well within expectation.^18^ The average GPC increases from 0.70 Å in the first 240 cycles to 0.81 Å in the next 180 cycles, and 1.2 Å in the final 180 cycles. The increase in GPC as the deposition progresses is a direct consequence of the transition from amorphous TiO_2_ growth to crystalline anatase growth, as it has been well documented that the GPC of ALD TiO_2_ is increased on anatase surfaces as compared to amorphous surfaces.^21−23^ This difference is the result of a higher density of hydroxyl groups present on the surface of anatase TiO_2_, which allows for more precursor adsorption per cycle.^23^

AFM measurements were performed to further study grain formation by looking at the surface topography (Figure 2d–f). The 240-cycle sample has already formed nuclei, which are randomly positioned and protrude up to 6 nm from their smooth surroundings (Figure S2). The fact that Raman and XRD studies do not reveal crystallinity indicates that the volume fraction of crystalline material is low. After 420 cycles, both the surface roughness and lateral grain size have increased. Each grain appears to enclose at least one dot with increased height. These dots most likely correspond with the nuclei observed for the 240-cycle sample. Besides lateral growth, from literature it is known that the grains also grow toward the substrate, crystallizing previously deposited amorphous TiO_2_.^32^ After 600 cycles, the height differences between the grains have increased, and where after 420 cycles the grains where pyramidal in shape, after 600 cycles the surface of each individual grain appears to be more flat (see also Figure S2 in the Supporting Information for AFM line profiles and Figure S3 for TEM sideview images).

#### Effect of Deposition Temperature

**Figure 3:**
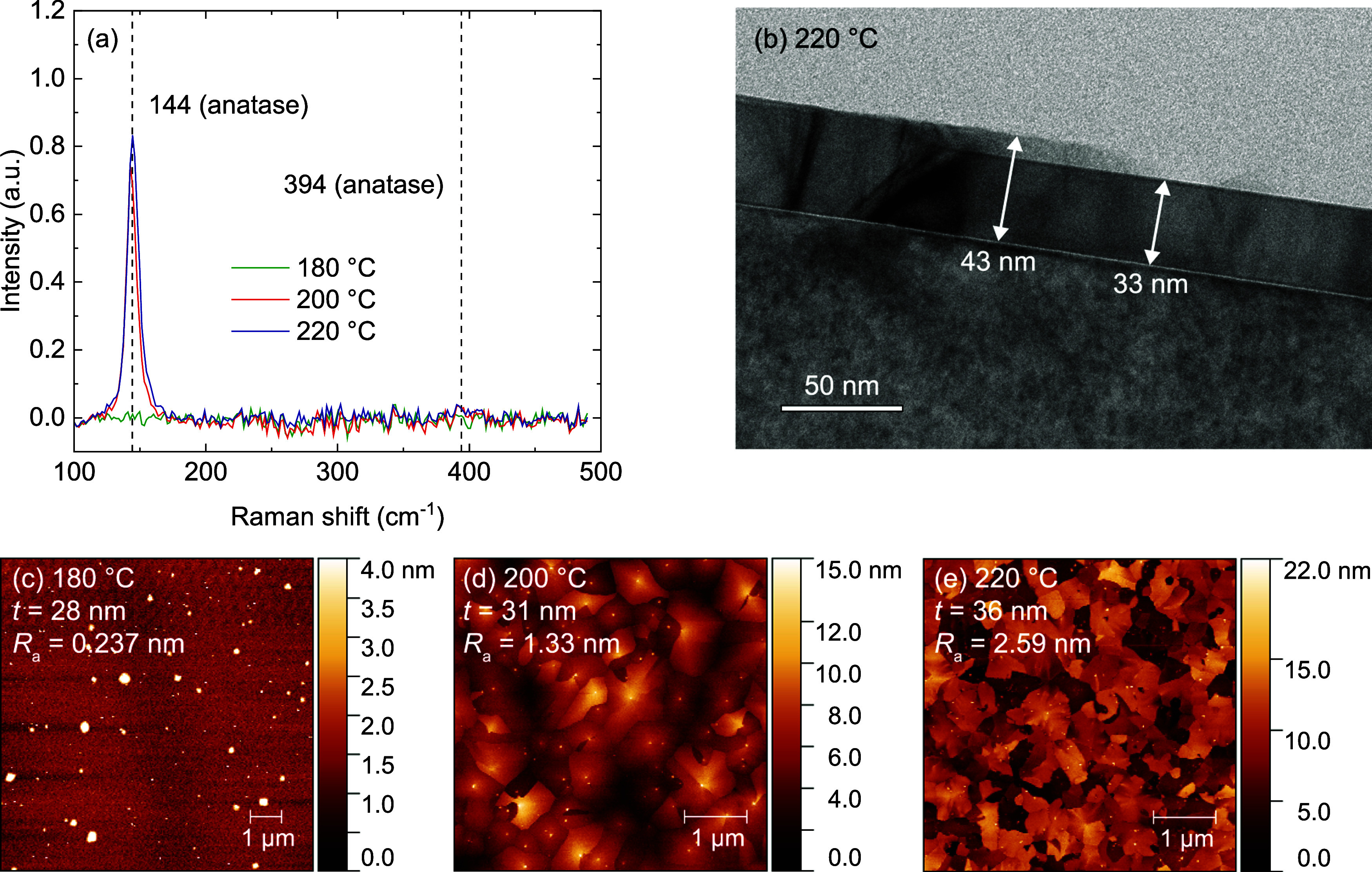
(a) Raman spectra normalized for film thickness, (b) cross-sectional TEM image, and (c–e) AFM images of PE-s-ALD TiO_2_ on planar Si substrates, deposited at 180, 200, and 220 °C and substrate rotation frequency of 2 rpm, for 420 ALD cycles. Heights of two distinct grains are indicated in (b) in red. Film thicknesses determined by spectroscopic ellipsometry are shown in (c–e).

The effect of temperature on the crystallization of PE-s-ALD TiO_2_ was studied by applying growth temperatures ranging 180 to 220 °C during depositions of 420 cycles at 2 rpm rotation frequency (Figure 3). The deposition at 180 °C resulted in a 28 nm film that appears amorphous from the Raman measurement. With AFM, however, the formation of protrusions at the surface were observed which indicate nuclei formation, similar to the 240-cycle sample grown at 200 °C discussed earlier. These results show that for a ∼30 nm TiO_2_ deposition, the critical deposition temperature for the amorphous-anatase phase transition is between 180 and 200 °C. At a growth temperature of 220 °C, the Raman signal increases only slightly, indicating that there is already enough energy at 200 °C for nearly all the amorphous material to be crystallized. The surface roughness increases (Figure 3c–e), similar to what was observed when increasing the number of cycles from 420 to 600 at 200 °C. Furthermore, the 220 °C sample is thicker than the 200 °C sample (i.e., 36 nm compared to 31 nm). These two observations indicate that the critical thickness for crystallization is reached earlier at elevated temperatures, as earlier crystallization leads to an earlier increase in GPC, and higher final film thickness and surface roughness. This trend will be shown later for depositions inside 3D substrates. Because of the large degree of crystallization of the 220 °C sample, this sample was selected for cross-sectional TEM analysis (Figure 3b). It can be observed that the grains indeed extend all the way to the substrate, as discussed earlier. Furthermore, a significant height difference of 10 nm can be observed between two adjacent grains that are present within the thickness of the TEM sample, the difference being similar to what is measured by AFM (Figure 3e).

#### Effect of Substrate Rotation Frequency

**Figure 4:**
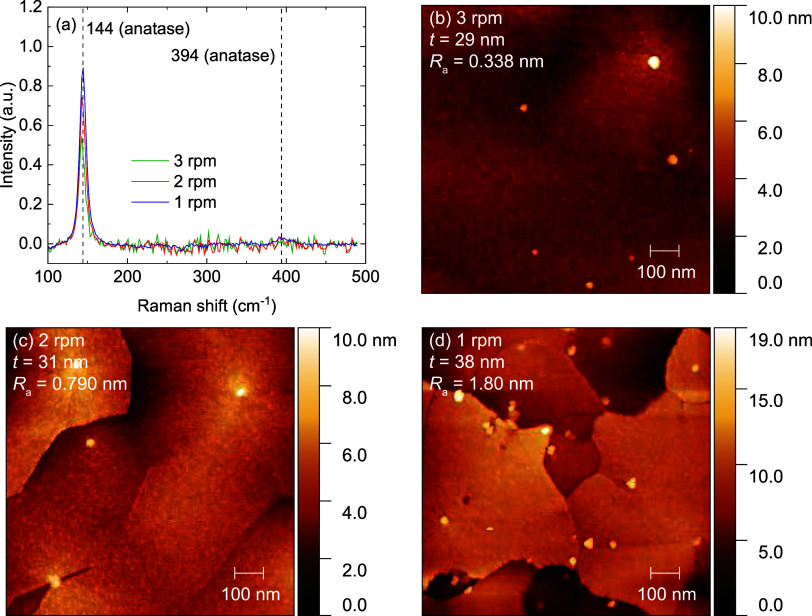
(a) Raman spectra normalized for film thickness, and (b–d) AFM images of PE-s-ALD TiO_2_ on planar Si substrates, deposited using substrate table rotation frequencies of 1, 2, and 3 rpm. Film thickness was determined by spectroscopic ellipsometry.

The effect of the substrate rotation frequency on the crystallinity of TiO_2_ was studied by performing 420 ALD cycles at 200 °C, with rotation frequencies of 3, 2, and 1 rpm (Figure 4). SE measurements showed the film thickness increasing from 29, to 31, to 38 nm as the rotation frequency is lowered, while Raman measurements show increased anatase crystallinity. Varying the rotation frequency of a rotary s-ALD reactor influences many different parameters simultaneously (e.g., precursor exposure time, precursor purge time, plasma exposure time, plasma purge time). Since the slow rotation frequencies used in this work already ensure the process to be well saturated, the most important parameter influenced by the rotation frequency is expected to be the total processing time. An annealing temperature of 200 °C has been shown to be sufficient to crystallize a mostly amorphous film containing only small nuclei to fully crystallize if the film is annealed long enough.^33^ Therefore, we hypothesize that the increased crystallization and GPC for reduced rotation frequency is the result of a higher thermal budget between each cycle. Additionally, an increased nuclei density is observed with AFM for the 1 rpm sample, with 30 ± 2 μm^–2^ for the 1 rpm sample, as compared to 7.4 ± 0.6 μm^–2^ for the 2 rpm sample (mean and standard deviation determined over 7 scans of 1 × 1 μm^2^). This increase could either be the direct result of increased plasma exposure, or the result of a longer total processing time.

### Deposition in Lateral High-Aspect-Ratio Structures

**Figure 5:**
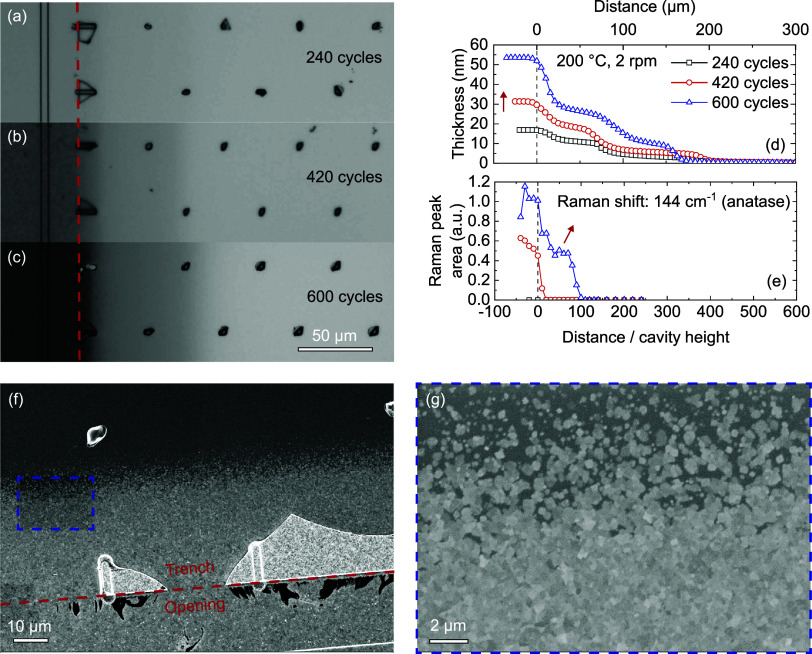
TiO_2_ films deposited in LHAR structures with varying number of ALD cycles. Optical microscopy images of (a) 240 cycles, (b) 420 cycles, and (c) 600 cycles TiO_2_. (d) Film thickness, and (e) Raman peak area normalized to film thickness as a function of distance in the structure. Trench openings are indicated with dashed lines and trends in thickness and Raman peak area are indicated by arrows. (f) SEM image of the start of the structure, with a close-up of the dashed square region shown in (g), which is the transition from anatase to amorphous.

After having analyzed the crystallization behavior on planar wafers, a similar study was performed on LHAR structures, starting by changing the number of cycles (Figure 5a–c). Relatively short plasma exposure times were used to guarantee the recombination-limited regime (i.e., plasma exposure and not precursor exposure is the limiting factor for the penetration depth (PD) of the film inside the structure). Thickness profiles of the deposited films were determined by performing SE line scans inside the structure (Figure 5d). Generally, for PE-ALD depositions inside 3D structures, a plateau (i.e., near-constant thickness) is expected up to a certain distance where the radicals are depleted.^34^ Here, the TiO_2_ depositions show two plateaus, as well as an additional plateau beyond the point where radicals are depleted. The latter is likely because of the presence of additional O-containing molecules (e.g., H_2_O or O_3_) inside the trench that can act as co-reactant. Since the conformality is limited by the plasma step, precursor molecules penetrate beyond the radical depletion point and bind to the surface. In the absence of any other O-source, the growth in this region stops after the surface is saturated with precursor molecules. However, here we observe continued growth. We hypothesize that other O-containing molecules are present, which react with the adsorbed precursor. Examples include water, which is a byproduct of the combustion reaction between the O_2_ plasma and the organic ligands at the surface, and O_3_ which is typically also formed in an O_2_ plasma. Trends in this region will be discussed in more detail in Effect of Temperature Section.

The difference in thickness between the film deposited outside and just inside the structure is due to two mechanisms. (1) The outside is directly exposed to UV photons generated in the N_2_–O_2_ plasma, while the rest of the structure is not in direct line-of-sight. Absorption of photons with energies above the band gap of TiO_2_ can photocatalytically enhance the growth rate of the ALD process.^24^ This effect occurs for both amorphous and anatase TiO_2_ but is more pronounced for the latter. Another effect that typically plays a role on directly exposed surfaces is the influence of ions on the growth per cycle.^35^ However, this effect can be excluded because of the atmospheric pressure, which leads to very low ion energies due to their many collisions.^36^ (2) The crystal phase has a large effect on the GPC, and anatase TiO_2_ is initially only present in the trench opening and slowly extends into the trench with increasing number of cycles (Figure 5e). In Figure 5f,5g the front of the anatase phase can be seen for the 600 cycles sample to be at a depth of about 30 μm. Here, the size and density of the grains decrease spatially over a few μm until only amorphous film is left. This front up to where crystallinity is observed by SEM and Raman lies deeper than the drop in film thickness observed around a depth of 10 μm. This is a logical consequence, since at this front of crystallization not a significant number of ALD cycles with enhanced growth rate on crystalline surfaces will have been performed.

#### Effect of Temperature

**Figure 6:**
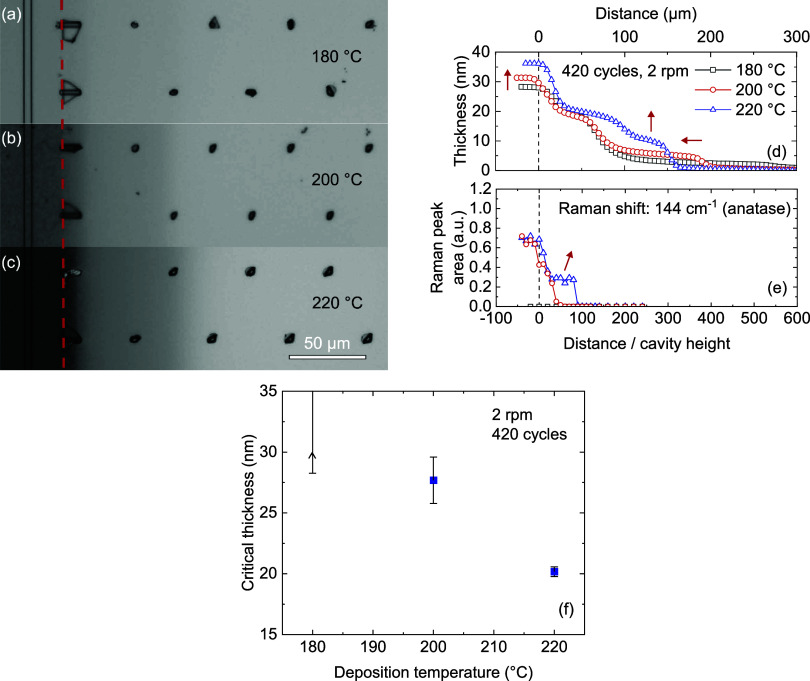
TiO_2_ films deposited in LHAR structures with varying deposition temperatures. (a–c) Optical microscopy images with the start of the trench indicated by the dashed line, (d) film thickness determined by spectroscopic ellipsometry, and (e) Raman peak area normalized to film thickness as a function of distance in the structure. Arrows indicate trends in film thickness and Raman peak area. (f) Critical film thickness for crystallization as a function of deposition temperature. The value at 180 °C is estimated to be larger than 30 nm.

The effect of deposition temperature on the thickness and crystallinity profiles is shown in Figure 6. The increase in crystallinity with temperature observed previously on planar substrates translates to crystallization deeper inside the structure on 3D substrates (Figure 6e). In turn, this has affected the thickness of the films in the trench opening, and the first 20 μm of the trench in the case of 220 °C, with larger thicknesses for higher temperatures (Figure 6d). Since the thickness gradually decreases in the trench, the critical film thickness for crystallization can be accurately determined from the thickness profiles (Figure 6f). Here, the critical thickness is defined as the smallest thickness where the anatase peak is observed in the Raman spectrum. Note that this is a larger thickness than required for initial grain formation, as can be seen from the AFM data, but before the point where the grains have expanded all the way down to the substrate interface. There is a clear drop in critical thickness with increasing temperature, which is as expected from a kinetics point of view.

**Figure 7:**
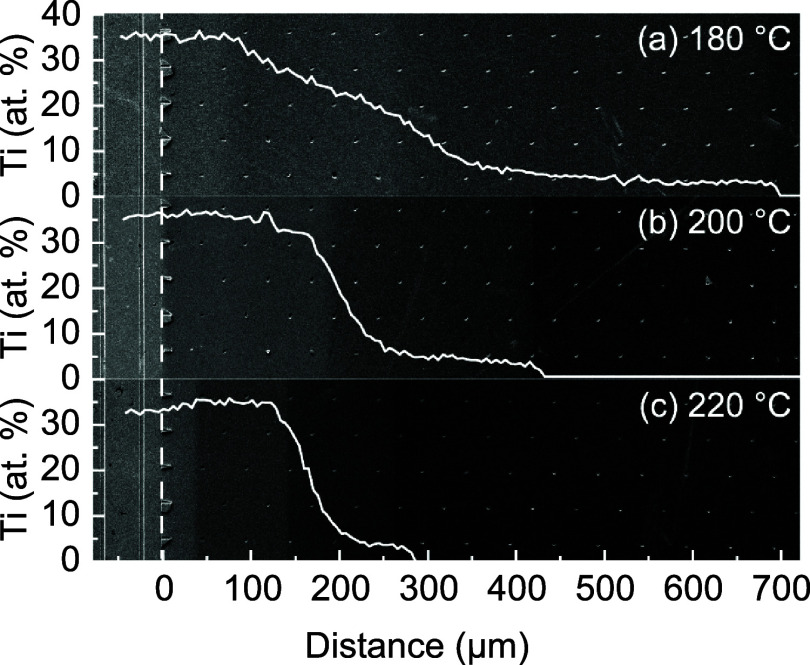
SEM image of TiO_2_ deposited inside LHAR structures at (a) 180 °C, (b) 200 °C, and (c) 220 °C, overlain with spatially resolved Ti content determined by XPS line-scan measurements. Dashed lines indicate the start of the trench.

Besides the changes near the trench opening, the temperature also influences film thickness deeper inside the trench, specifically in the region where O radicals are depleted. As discussed earlier, other O-containing molecules (e.g., H_2_O or O_3_) are hypothesized to be present here and result in TiO_2_ deposition with a significantly lower GPC. The film thickness in this region increases with temperature, while the penetration depth (PD) decreases. These trends can be seen more clearly when using surface sensitive techniques such as SEM and XPS (Figure 7). The decrease in PD with increasing temperature suggests the presence of O_3_, since the decomposition rate of O_3_ strongly increases with temperature.^37^

#### Effect of Substrate Rotation Frequency

**Figure 8:**
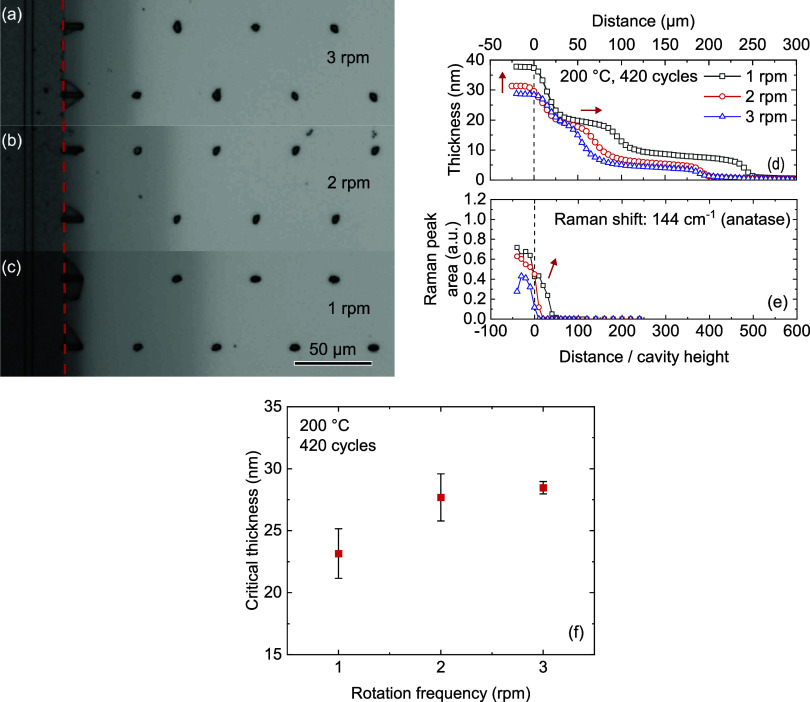
TiO_2_ films deposited in LHAR structures with varying rotation frequencies of the substrate table. (a–c) Optical microscopy images with dashed lines indicating the start of the trench. (d) Film thickness determined by spectroscopic ellipsometry. (e) Raman peak area normalized to film thickness as a function of distance in the structure. Arrows indicate trends in thickness and Raman peak area. (f) Critical film thickness for crystallization as a function of rotation frequency.

The influence of substrate rotation frequency on film thickness and crystallinity is shown in Figure 8. The rotation frequency of the s-ALD reactor is directly linked to the dose and purge times of both the precursor and plasma. The most significant effect of lowering the rotation frequency of these recombination-limited depositions is therefore the increase in the PD, since *PD* ∝ *ln*(*plasma dose*).^34^ The obtained PDs for the various rotation frequencies will be used in the discussion section to determine the recombination probability of the oxygen radicals.

Lowering the rotation frequency also appears to promote crystallization, as the Raman signal emanates from deeper inside the structure. While many parameters are directly influenced by the rotation frequency, as mentioned above, the biggest contributor to this effect is expected to be the processing time. With a reduced rotation frequency, the film spends more time per cycle at the processing temperature of 200 °C, which is a sufficiently high temperature to promote grain growth.^33^ This extended thermal anneal results in a reduced critical thickness with decreasing rotation frequencies (Figure 8f).

## Discussion

The presented results show a myriad of effects in the growth and crystallization of TiO_2_ on 3D substrates: partial crystallization, enhanced growth on the crystalline film, UV-enhanced growth, and additional growth beyond the point of radical depletion due to the presence of other O-sources. These effects are expected to reduce the feasibility of conformal crystalline film depositions for TiO_2_-based technologies with aspect ratios of a few tens and upward, such as supported and immobilized catalysts.

From the presented results and previous reports in the literature, we provide a comprehensive overview of the TiO_2_ crystallization and growth propagation in LHAR structures in this section. Furthermore, the presented results are used to determine the oxygen radical recombination probability at atmospheric pressure and 200 °C. This effort expands on previous work, where recombination probabilities were determined for atmospheric-pressure PE-s-ALD at 100 °C, as well as at various temperatures for low-pressure temporal ALD.^9,25^

### TiO_2_ Crystallization and Growth Propagation in LHAR Structures

**Figure 9:**
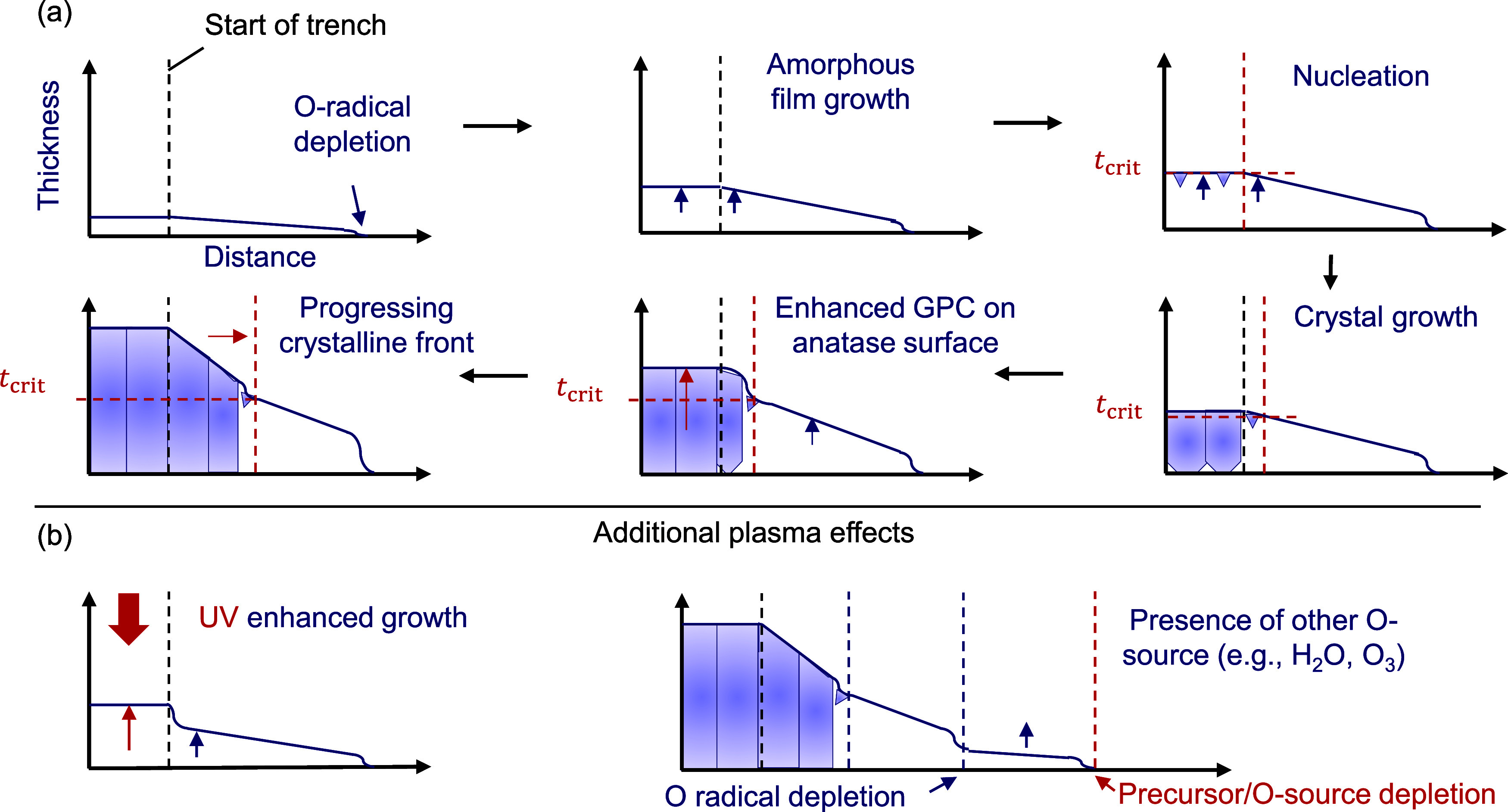
Schematic depiction of the influence of crystallization on conformality of TiO_2_ ALD inside a LHAR structure. (a) Growth and crystallization mechanism. Black dashed line represents the start of the trench, red dashed horizontal and vertical lines represent the critical film thickness for nucleation, and the crystallization front inside the trench, respectively. (b) Additional effects that occur in the case of PE-s-ALD. Note that thickness is typically in the nm range, while distance is typically in the μm range.

A schematic overview of ALD TiO_2_ growth and crystallization inside a LHAR structure is shown in Figure 9. The deposition starts off with a purely amorphous film, of which the thickness slightly decreases with respect to position in the structure until one of the reactants (typically oxygen radicals in the case of PE-ALD) is fully depleted. This gradual thickness decrease is commonly observed in high-aspect-ratio structures, both for thermal and plasma ALD.^34,38,39^ At some point during the deposition, the film thickness outside the structure reaches the critical film thickness for nucleation of crystals. The reason films crystallize once the critical thickness is reached might be due to the accumulation of growth stress.^19^ The experimentally observed critical thickness is not to be confused with the thermodynamic critical thickness. The thermodynamic critical thickness is the thickness where the increased surface energy from a crystalline surface compared to an amorphous surface is compensated by the reduced bulk energy from a crystal lattice compared to amorphous material, so that Δ*G*_crystallization_ = 0. The experimentally observed critical thickness is offset toward larger thicknesses because of the activation energy of the phase transition.^40^ Next, the grains will grow by transforming the surrounding amorphous material, both laterally, as well as down toward the substrate.^32^ In the crystalline region, subsequent cycles will experience enhanced GPC because of the higher density of hydroxyl groups present at the anatase surface.^23^ As the deposition proceeds, the crystallization front progresses further into the structure, as an expanding region of the film exceeds the critical thickness. This mechanism is expected to be universally true for both thermal and plasma-enhanced ALD. However, when using plasma, two additional effects can be observed (Figure 9b). First, UV photons generated in the N_2_–O_2_ plasma can enhance the growth of film areas in direct line-of-sight from the source. Second, there is film growth beyond the depletion point of oxygen radicals due to the presence of other O sources such as water or O_3_, as discussed in Effect of Temperature Section.

This work has several implications for the application of TiO_2_ on 3D structures. In order to obtain perfect step coverage, different processing conditions should be used depending on the desired morphology. Near perfect step coverage of amorphous TiO_2_ can be easily obtained by using low deposition temperatures, where limiting UV exposure to the entirety of the substrate is expected to help prevent local enhanced growth. For applications that require near perfect step coverage of anatase crystalline films, a thermal annealing step can be incorporated in the process once the critical film thickness is reached throughout the structure before continuing the deposition. This allows for crystallization throughout the entire film, ensuring the increased growth rate of anatase material throughout the entire film for the remainder of the deposition.

### Oxygen Radical Recombination Probability

The depositions performed at varying rotation frequencies can also be used to determine the recombination probability of oxygen radicals during the process. This parameter is important for PE-ALD in 3D structures, as the radical dose is typically limiting for the conformality. The recombination probability is therefore a useful indicator for how easily a material can be conformally deposited using PE-ALD.^34^ For example, conformal depositions of Al_2_O_3_ and HfO_2_, which have recombination probability values of 10^–3^–10^–1^, are notoriously difficult in high-aspect-ratio structures, while conformal depositions of SiO_2_ and TiO_2_ with values of 10^–5^–10^–4^, are relatively straightforward. Determining the recombination probability can be done using a method based on a reaction-diffusion model, which describes the PD to scale logarithmically with the radical dose.^34^ Observing the change in PD as a function of the plasma exposure time therefore allows for extraction of the recombination probability. In our previous work, we determined the scaling of the PD to be
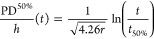
for atmospheric pressure conditions and trench-shaped features with a critical feature size of 500 nm, as also used in this work.^9^ Here, PD^50%^ is the 50%-thickness penetration depth, *h* is the height of the cavity, *t* is the dosing time, *r* is the recombination probability, and *t*_50%_ is in approximation the dosing time required to reach 50% saturation on a planar substrate.

**Figure 10:**
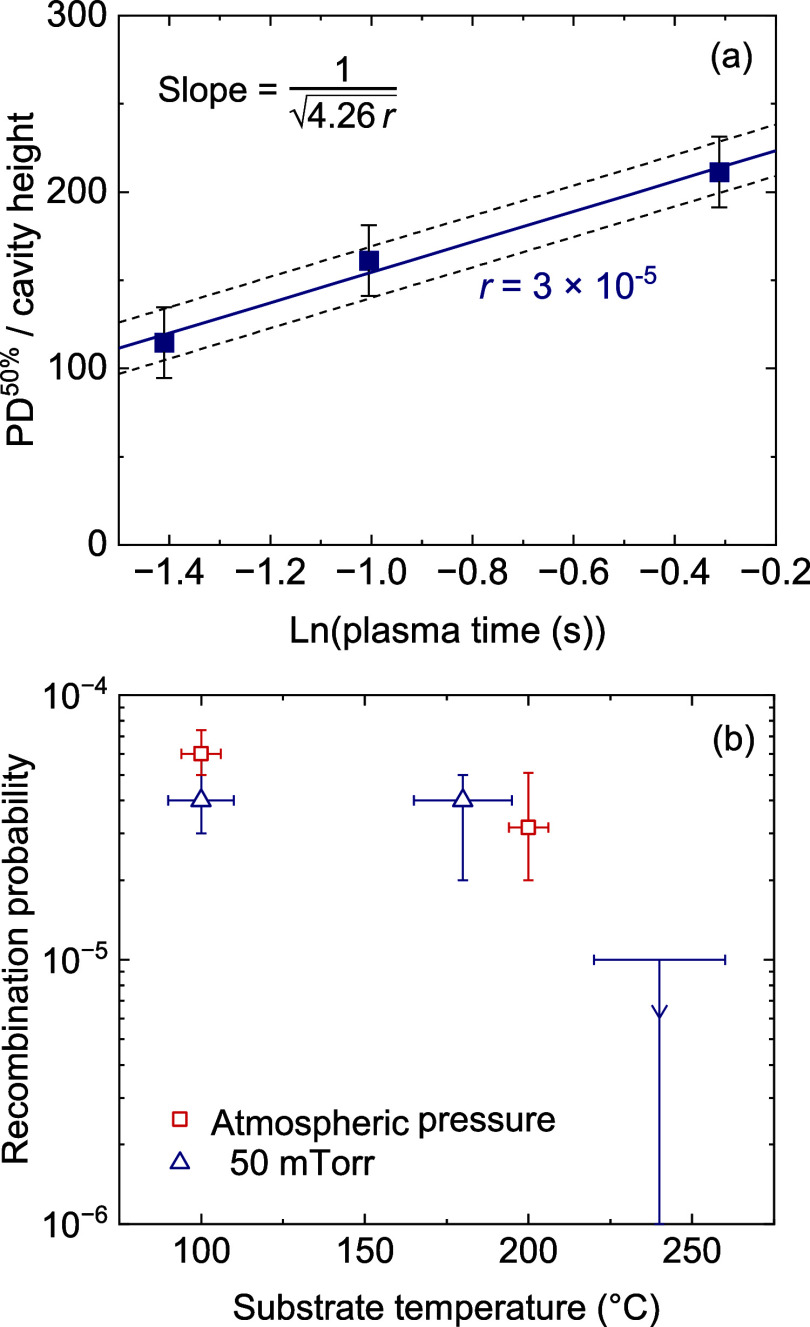
(a) The 50%-thickness penetration depth scaled to cavity height as a function of the plasma exposure time from which the recombination probability was extracted. (b) Extracted oxygen radical recombination probability at 200 °C at atmospheric pressure plotted together with previous data at 100 °C, and at various temperatures at 50 mTorr.^9,25^

The PD^50%^ scaled to cavity height was plotted against the natural logarithm of the plasma exposure time (Figure 10a) The recombination probability was determined to be 3 × 10^–5^ (uncertainty range: 2–5 × 10^–5^) at 200 °C from the slope of the linear fit through the data points. Figure 10b shows the recombination probability data of this work along with data obtained with the same method at 100 °C at atmospheric pressure, as well as several temperatures at low pressure (i.e., 50 mTorr).^9,25^ The recombination probability is slightly lower at 200 °C than at 100 °C. A likely explanation is that, at these conditions, the probability of impinging radicals to recombine with species adsorbed to the TiO_2_ surface (e.g., O and O_2_) is limited by the density of these species. Therefore, the probability drops as the desorption rate increases with temperature. On the other hand, the influence of pressure appears to be very small, as such a large difference in pressure yields values with the same order of magnitude. The trend of reduced recombination probability at increasing temperatures is also consistent across the different pressures, although more data points are required to see if the onset temperature for this decrease is also the same. All-in-all, differences in conformality between low-pressure and atmospheric-pressure PE-ALD are not caused by a difference in recombination probability, but rather by differences in initial radical density, diffusion behavior, and number of collisions.^9,41^

## Conclusions

This work presents a systematic study of PE-s-ALD anatase TiO_2_ on planar and 3D substrates. A mechanism for the growth and crystallization inside 3D structures is presented, highlighting an interplay between four different effects: (1) enhanced growth in the trench opening because of direct exposure to UV photons, (2) gradual decrease of thickness inside the structure resulting in partial crystallization, (3) subsequent enhanced growth of crystalline film at the structure opening, and (4) subtle growth beyond the depletion of oxygen radicals due to the presence of O_3_. Similar mechanisms might occur for other materials which also start to crystallize after reaching the critical film thickness, such as In_2_O_3_, HfO_2_, ZrO_2_, and Hf_*x*_Zr_1–*x*_O_2_,^42−45^ although future research will be required to confirm this. Finally, the analysis of radical recombination probabilities reveals major similarities with low-pressure conditions, including decreasing values for increasing temperatures. This result suggests that the initial reactant partial pressure and the diffusion behavior are the only major contributions to differences in conformality between atmospheric-pressure and low-pressure PE-ALD. Overall, this research contributes to gaining precise control over crystallinity and conformality during high-throughput depositions of TiO_2_ thin films using PE-s-ALD.

## Supporting Information Available

Gonio XRD data, AFM line scan profiles, and sideview TEM images of the deposited TiO_2_ films (PDF)
