# Supplementary material for: Plasma-Enhanced Spatial Atomic Layer Deposition on 2D and 3D Surface Topologies: The Case of Amorphous and Crystalline TiO2
Source: J Phys Chem C Nanomater Interfaces. 2025 Jan 31;129(6):3310–20. doi: 10.1021/acs.jpcc.4c08281 (PMC11831676; doi:10.1021/acs.jpcc.4c08281)
Supplement: Supplementary file 1 — jp4c08281_si_001.pdf [file jp4c08281_si_001.pdf]

# Plasma-Enhanced Spatial Atomic Layer Deposition on 2D and 3D Surface Topologies: The Case of Amorphous and Crystalline $TiO_2$

Mike van de Poll [1], Jie Shen [2], James Hilfiker [3], Marcel Verheijen [1], Paul Poodt [1,4], Fieke van den Bruele [2], Wilhelmus Kessels [1], Bart Macco [1,*]

[1] *Department of Applied Physics and Science Education, Eindhoven University of Technology, 5600 MB Eindhoven, The Netherlands*

[2] *TNO/Holst Centre, High Tech Campus 31, 5656 AE Eindhoven, The Netherlands*

[3] *J.A. Woollam Co., Inc., 311 South 7th Street, Lincoln, Nebraska 68508, USA*

[4] *SparkNano B.V., Esp 266, 5633 AC Eindhoven, The Netherlands*

** Email: b.macco@tue.nl*

## Supporting information

**Additional $TiO_2$ microstructure analysis**

Additional analysis of XRD, AFM, and TEM measurements was performed to discern preferred crystal orientation and crystal shape. The grazing-incidence XRD (GI-XRD) measurements in the main text show the crystallization as function of film thickness. The GI-XRD data show a limited subset of peaks expected for a film with randomly oriented crystals. The asymmetrical detection geometry complicates interpretation about preferred crystal orientation. For this purpose, a gonio XRD measurement was performed on the 54 nm thick $TiO_2$ film and compared to anatase $TiO_2$ powder data, [1] The latter shows the relative intensity of all possible reflections in case of a randomly oriented film, which is characteristic for a powder, containing randomly orientated crystals (Figure S1**Error! Reference source not found.**). The data is normalized by scaling of the (101) peaks to the same intensity. This comparison clearly shows that <101> is the preferred crystal orientation.

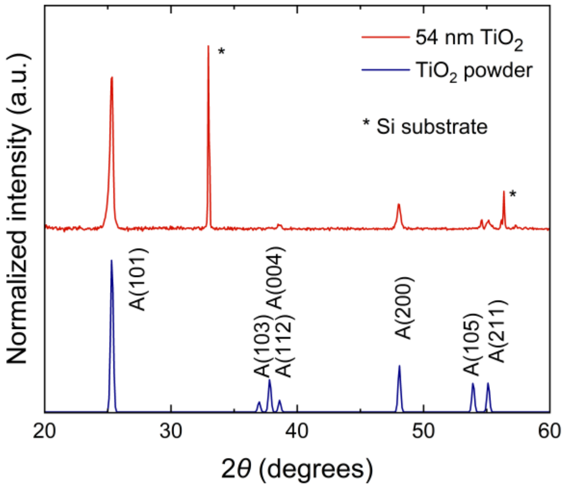


Figure S1: Gonio XRD data of 54 nm $TiO_2$ (600 cycles) along reference $TiO_2$ powder data from Horn *et al.* [1]

From AFM images in the main text (Figure 2d-f) it looks like the surface topography of the grains is changing with the number of cycles. This transformation was confirmed by plotting line profiles for randomly chosen horizontal lines in these AFM images (Figure S2). After 420 cycles (Figure S2b), the grains which have formed around the nuclei appear to have pyramid-shaped top surfaces, while the grains after 600 cycles (**Error! Reference source not found.**c) appear to have more flattened surfaces.

In each line scan, at least one sharp peak can be seen, with a height up to 6 nm, which corresponds to a nucleus. Throughout all the AFM images in the main text it can be observed that the nuclei density increases with temperature, as well as with the number of cycles, and the exposure times.

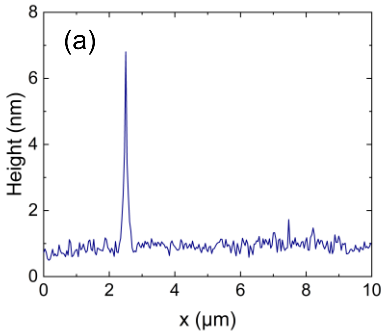


Figure S2: AFM line profiles corresponding to horizontal lines in AFM images Fig. 2d-f in the main text, corresponding to (a) 240 cycles, (b) 420 cycles, and (c) 600 cycles of $TiO_2$.

Sideview TEM analysis was performed on the sample deposited at 220 °C (Figure S3). The film is entirely crystalline. From the top surface roughness, two potential locations of original nucleation were observed, with grains starting midway through the height of the film, with different crystalline orientations than the rest of the layer. Note that the origins of these grains are unknown, as they could be the remaining parts of larger grains that were present before preparation of the thin TEM samples.

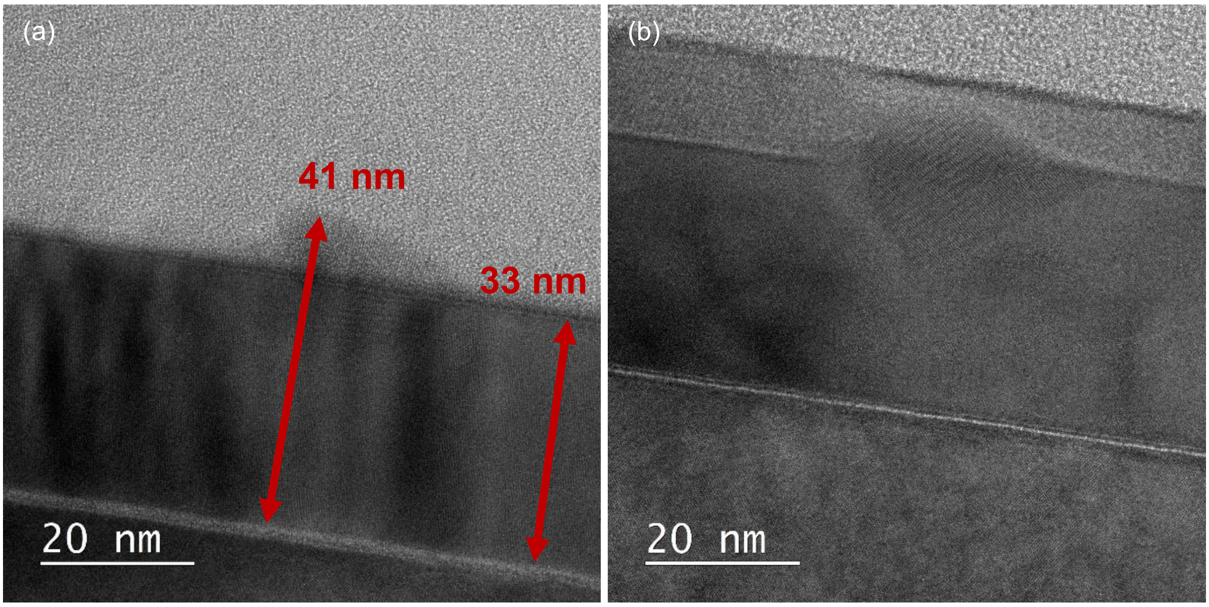


Figure S3: Sideview TEM images of $TiO_2$ deposited at 220 °C using 420 cycles at 2 rpm rotation frequency. Arrows indicate the locations at which film thicknesses were determined.

1. Horn, M., Schwerdtfeger, C. F. & Meagher, E. P. Refinement of the structure of anatase at several temperatures. *Zeitschrift fur Kristallographie - New Crystal Structures* **136**, 273–281 (1972).
